# A three-component dynamical index of consciousness-related neural organisation

**DOI:** 10.1007/s00422-026-01049-1

**Published:** 2026-07-13

**Authors:** Hassan Ugail, Newton Howard

**Affiliations:** 1 Centre for Visual Computing and Intelligent Systems, University of Bradford, Bradford, UK; 2 School of Individualized Study, Rochester Institute of Technology, Rochester, USA

## Abstract

Quantifying consciousness from brain activity remains a major challenge in neuroscience and clinical practice. Many existing EEG measures focus on a single feature of neural activity, such as complexity, synchrony, or spectral structure, but no single feature appears sufficient across different brain states. We introduce a composite dynamical framework that combines three complementary properties of brain activity, i.e., scale-free temporal organisation, cross-frequency organisation, and metastable flexibility in large-scale synchronisation. These components are normalised and combined into a single index designed to capture organised dynamical complexity rather than raw signal complexity alone. We test the framework in both synthetic and empirical settings. In a generative model of nine EEG-like brain states, including wakefulness, dreaming, anaesthesia, non-conscious states, and seizure states, the index separates the synthetic conscious and non-conscious classes without overlap and remains stable across ablation, sensitivity, and Monte Carlo analyses. We then apply the framework to two-channel Sleep-EDF recordings from 30 healthy adults, where it provides a proof-of-principle subject-level separation of wakefulness from N2 and REM sleep. The framework is dynamical-systems-inspired and is not committed to any single theory of consciousness, making it compatible with a range of theoretical perspectives. With further validation, the framework may be applicable across multichannel brain recordings, including anaesthesia, disorders of consciousness, and basic consciousness-research settings.

## Introduction

The scientific study of consciousness aims to bridge subjective experience with the objective dynamics of brain activity. Significant progress has been made in identifying neural correlations of conscious perception and state transitions, yet a general, mechanistically interpretable, and robustly validated index of conscious level remains elusive. Leading theories, such as Global Neuronal Workspace Theory (GNWT) and Integrated Information Theory (IIT), describe consciousness in terms of large-scale information broadcasting and integrated causal structure, respectively (Dehaene 2014; Tononi et al. 2016). Recurrent Processing Theory and higher-order theories emphasise the roles of feedback interactions and metarepresentational mechanisms (Lamme 2020; Lau and Rosenthal 2011). Meanwhile, dynamical systems approaches highlight metastability, criticality, and multiscale complexity as fundamental hallmarks of conscious brain function (Tognoli and Kelso 2014; He 2010; Sarasso et al. 2021).

Empirically, numerous quantitative measures of neural complexity, connectivity, and integration have been proposed. Perturbational complexity indices derived from transcranial magnetic stimulation and EEG have shown promise as state-independent markers of consciousness capacity (Casali et al. 2013; Nilsen et al. 2024). Spontaneous EEG and MEG complexity measures differentiate wakefulness, sleep, anaesthesia, and psychedelic states (Schartner et al. 2015; Carhart-Harris et al. 2014; Montupil et al. 2023). Cross-frequency coupling (CFC), especially theta–gamma phase–amplitude coupling, is linked to memory and higher cognitive functions (Canolty and Knight 2010; Siebenhühner et al. 2020; Muthuraman et al. 2020; Musaeus et al. 2020). However, known pitfalls in its estimation must be carefully addressed to avoid artefactual results (Aru et al. 2015). Alterations in CFC are observed in neurodegenerative and psychiatric disorders (Benetton et al. 2025). Metastability measures, associated with flexible cognitive control, are reduced in pathological and unconscious states (Tognoli and Kelso 2014; Hancock et al. 2023; Wijaya et al. 2025).

Despite these advances, the field confronts several challenges. Many existing metrics capture only a single dimension of dynamics—such as global connectivity, entropy, or spectral power—and fail to integrate multiscale structure, cross-frequency organisation, and metastable transitions explicitly. Even when individual measures perform well in specific contexts, their generalisability across paradigms and recording modalities remains uncertain (Casey et al. 2024; Liu et al. 2023). Established single-channel markers such as the spectral slope (Lendner et al. 2020; Brake et al. 2024) and Lempel–Ziv complexity (Höhn et al. 2024) perform well for some state contrasts, but may fail for others, motivating the development of complementary multidimensional frameworks. Recent adversarial collaborations comparing GNWT and IIT highlight the need for reproducible and falsifiable methodologies (Doerig et al. 2021; Melloni et al. 2023; Ferrante et al. 2025; Luppi et al. 2024). Large-scale screening efforts further highlight the value of computationally efficient, multi-feature EEG markers for disorders of consciousness (Sitt et al. 2014). Consequently, there is a clear demand for metrics that are both theoretically grounded and empirically robust.

This paper addresses this need by introducing a multidimensional framework for quantifying consciousness-related dynamics. Instead of seeking a single scalar measure of consciousness, we decompose neural organisation into three dynamical properties consistently emphasised across theories, i.e., scale-free temporal organisation (*H*), organised cross-frequency complexity (*D*), and metastability (*M*). Scale-free temporal organisation reflects long-range coordinated activity across temporal scales, characterised by fractal-like fluctuation structure that extends beyond independent local activity. Organised complexity captures structured phase–amplitude relationships between oscillatory components and temporal signal richness. Metastability quantifies the variability of large-scale synchronisation over time, indicating the coexistence of integration and segregation.

Since consciousness is not directly observable, validation requires a two-stage strategy. First, we develop a generative model for synthetic EEG-like data that incorporates ground-truth manipulations of scale-free temporal organisation, cross-frequency coupling, and metastability. This approach builds on prior simulation-based benchmarking of complexity measures and causal connectivity estimators (Schreiber and Schmitz 2000; Barnett and Seth 2015; Schartner et al. 2015). Second, we compute the proposed metrics on these synthetic signals and evaluate their ability to discriminate between states with known dynamical organisation. Critically, we then validate the framework using real EEG from 30 Sleep-EDF subjects (1,078 artefact-free epochs). We benchmark our index Ψ against three established markers (Lempel–Ziv complexity, spectral slope, and alpha power) on the same data, using the subject-level unit of analysis with 95% bootstrap confidence intervals for all AUC estimates. A matched-pairs Friedman omnibus with Bonferroni-corrected pairwise Wilcoxon tests and rank-biserial effect sizes provides statistical evaluation that properly accounts for within-subject dependence.

Our framework is dynamical-systems-inspired. It does not adjudicate between GNWT, IIT, or other accounts, nor does it presuppose any specific metaphysical stance on subjective experience. Instead, it formalises structural motifs widely regarded as necessary for conscious-level processing and provides a toolset for quantifying these motifs in both simulated and empirical data.

## Background and theoretical context

The conceptual foundations of this framework integrate several research strands. First, the relationship between complexity and consciousness suggests that conscious states require both integration and differentiation, preventing brain activity from fragmenting into independent components while maintaining high informational content (Balduzzi and Tononi 2009; Tononi et al. 2016; Seth et al. 2011). Empirical studies show that EEG complexity decreases during propofol anaesthesia and non-rapid eye movement sleep (Schartner et al. 2015; Sarasso et al. 2021), increases during psychedelic states (Carhart-Harris et al. 2014; Montupil et al. 2023), and correlates with residual awareness in disorders of consciousness (Liu et al. 2023). Perturbational complexity indices further indicate that consciousness capacity declines when effective connectivity is disrupted by anaesthesia or deep sleep (Massimini et al. 2005; Casali et al. 2013; Nilsen et al. 2024). Recent reviews consolidate these findings, linking complexity, differentiation, and the level of consciousness (Sarasso et al. 2021).

A second strand concerns cross-frequency coupling. Neural oscillations at different frequencies interact crucially in perception, memory, and decision-making (Canolty and Knight 2010; Siebenhühner et al. 2020; Muthuraman et al. 2020; Musaeus et al. 2020). For example, theta–gamma phase–amplitude coupling in the hippocampus and neocortex supports working memory and episodic recall, with disruptions observed in ageing and neurodegeneration (Musaeus et al. 2020; Benetton et al. 2025; Al Qasem et al. 2024). We note that careful estimation of CFC, including surrogate-based validation, is essential to avoid volume-conduction and signal-processing artefacts (Aru et al. 2015). Thus, CFC may serve as a mechanistic bridge between local computation and large-scale integration, making it a natural candidate for consciousness-related metrics.

A third strand focuses on metastability and criticality. Metastability, originally from physics and chemistry (Kuramoto 1984; Rossi et al. 2025), describes the brain’s tendency to balance integration and segregation (Tognoli and Kelso 2014; Hancock et al. 2025). Empirical studies show reduced metastability during anaesthesia-induced unconsciousness, severe brain injury, and in psychiatric conditions like schizophrenia (Hancock et al. 2023; Wijaya et al. 2025). Related work on critical dynamics suggests that the brain operates near a critical point, with scale-free fluctuations, and that deviations from criticality correlate with altered conscious levels (He 2010; Bonhomme et al. 2019; Maschke et al. 2024). These findings motivate including metastability as a core component of any quantitative framework for conscious dynamics.

Finally, the theoretical landscape is evolving. GNWT and IIT, once pursued independently, are now being directly compared through large-scale adversarial collaborations that prioritise preregistration, shared datasets, and theory-agnostic analyses (Melloni et al. 2023; Doerig et al. 2021; Ferrante et al. 2025; Luppi et al. 2024). These initiatives highlight the need for mathematically grounded, flexible metrics applicable across theoretical contexts. Our framework, centred on scale-free temporal organisation, cross-frequency organisation, and metastability, aims to contribute to this methodological ecosystem.

## Theoretical positioning and scope

This framework operates at the level of large-scale dynamical structure and deliberately avoids metaphysical commitments regarding consciousness. We treat consciousness as a latent property, hypothesised to be reflected in specific neural activity patterns. The central claim is not that the composite index Ψ measures consciousness directly, but that it tracks neural organisational aspects associated with conscious-level processing across multiple theories.

This stance yields several implications. First, the framework is *dynamical-systems-inspired*, and the choice of scale-free temporal organisation, cross-frequency coupling, and metastability as core components embeds a dynamical-systems ontology. GNWT can interpret large-scale integration and metastability as supporting global broadcasting and flexible workspace dynamics (Dehaene 2014; Mashour et al. 2020; Deco et al. 2021; Bonhomme et al. 2019). IIT can view integration and complexity as proxies for irreducible cause–effect structure (Balduzzi and Tononi 2009; Tononi et al. 2016). Dynamical systems perspectives can regard metastability and CFC as mechanisms for coordinating distributed computations (Tognoli and Kelso 2014; Hancock et al. 2025; Rossi et al. 2025). Second, by focusing on dynamical motifs rather than specific implementations, the framework can be applied across modalities, species, and potentially artificial systems, given suitable time-series data.

We focus on EEG-like signals for concreteness. EEG provides excellent temporal resolution and is widely used in consciousness research, anaesthesiology, and the assessment of disorders of consciousness (Giacino et al. 2018; Bonhomme et al. 2019; Casey et al. 2024; Liu et al. 2023). However, the metrics are defined generically and could be computed from MEG, intracranial EEG, or fast optical recordings with minimal modification. Application to slower modalities like fMRI would require adjustments, particularly in frequency-domain analysis, but the core principles of scale-free temporal organisation, cross-frequency structure, and metastability remain relevant.

We emphasise that this work focuses on methodological and conceptual development, not immediate clinical translation. While we discuss potential applications in anaesthesia monitoring, disorders of consciousness, and epilepsy, these require extensive empirical validation beyond our synthetic environment. We provide a proof-of-principle that a compact set of dynamical metrics can reproduce qualitative distinctions between states central to consciousness research.

No single resting-state metric currently captures the multiscale, multi-frequency, and dynamical properties jointly characteristic of conscious brain activity. Existing approaches often isolate one dimension. Perturbational complexity quantifies causal propagation but requires external perturbation; multiscale entropy captures scale-free structure but not functional organisation; cross-frequency metrics quantify coordination but not dynamical flexibility. Our framework addresses this gap by integrating three complementary components, i.e., (i) scale-free temporal organisation across temporal scales, (ii) organised cross-frequency complexity, and (iii) metastable dynamical richness. The resulting composite index Ψ reflects features consistently linked to conscious wakefulness, yet is general enough to compare diverse physiological, pharmacological, and pathological states.

## Mathematical framework

### Multivariate dynamical system

We model EEG activity as a multivariate stochastic process $X_t \in \mathbb {R}^C$ for t=1,⋯,T, where *C* is the number of channels. For any subset $S\subseteq \{1,\dots ,C\}$, the subsystem activity is $X_t^S = (X_{t,i})_{i\in S}\in \mathbb {R}^{|S|}$. In the synthetic experiments below, we compute metrics on the full set of channels, but the framework generalises to data-driven subsystem selection based on anatomical or functional criteria.

### Scale-free temporal organisation ($H_{\textrm{eff}}$)

Scale-free temporal organisation could be defined as the sum across scales of differences between whole-subsystem entropy and the sum of part entropies. Direct multi-dimensional entropy estimation is computationally demanding, so we approximate this property using detrended fluctuation analysis (DFA), which quantifies long-range correlations (Peng et al. 1994; He 2010).

For each channel *i*, consider the demeaned signal $x_i(t) = X_{t,i} - \bar{X}_i$ and its cumulative sum,

1 $$\begin{aligned} Y_i(k)=\sum _{t=1}^{k}x_i(t). \end{aligned}$$

We divide $Y_i$ into windows of length *s* across scales *s*, fit a linear trend per window, compute the root-mean-square deviation of detrended residuals, and average across windows to obtain the fluctuation function $F_i(s)$. If $F_i(s) \sim s^{\alpha _{\textrm{dfa},i}}$, then $\alpha _{\textrm{dfa},i}$ is the DFA scaling exponent. Values $\alpha _{\textrm{dfa}}\in (0.5,\,1.0)$ indicate long-range persistent correlations (fGn-like regime), $\alpha _{\textrm{dfa}}<0.5$ anti-persistent behaviour, $\alpha _{\textrm{dfa}}=0.5$ uncorrelated fluctuations, and $\alpha _{\textrm{dfa}}>1.0$ non-stationary, fBm-like dynamics.

The channel-averaged DFA scaling exponent is,

2 $$\begin{aligned} \bar{\alpha }_{\textrm{dfa}}=\frac{1}{C}\sum _{i=1}^{C} \alpha _{\textrm{dfa},i}. \end{aligned}$$

Since both uncorrelated fluctuations and excessive persistence are suboptimal for conscious-level processing, we transform $\bar{\alpha }_{\textrm{dfa}}$ using a tuning function that peaks at an optimal scaling exponent. In the synthetic analyses, we use a Gaussian tuning function,

3 $$\begin{aligned} H_{\textrm{eff}}^{\textrm{syn}}=\exp \!\left[ -\frac{\bigl (\bar{\alpha }_{\textrm{dfa}}- H_{\textrm{opt}}\bigr )^2}{2\sigma _H^2}\right] , \end{aligned}$$

with $H_{\textrm{opt}}=0.35$ and $\sigma _H=0.12$, consistent with the typical $\alpha _{\textrm{dfa}}$ regime of the Kuramoto-based synthetic ensemble (Section 5). For real EEG, to avoid sensitivity to between-subject variability in the Wake reference level, we adopt a range-normalised triangular tuning function,

4 $$\begin{aligned} H_{\textrm{eff}}^{\textrm{real}} = \max \!\left( 0,\,1 - \frac{|\bar{\alpha }_{\textrm{dfa}} - H_{\textrm{opt}}^{\textrm{subj}}|}{\Delta \alpha ^{\textrm{subj}}}\right) , \end{aligned}$$

where $H_{\textrm{opt}}^{\textrm{subj}}$ is the per-subject median of $\bar{\alpha }_{\textrm{dfa}}$ computed over that subject’s Wake epochs only, and $\Delta \alpha ^{\textrm{subj}}$ is the per-subject empirical range of $\bar{\alpha }_{\textrm{dfa}}$ across all retained epochs for that subject (with a global-range fallback when the per-subject range is below 0.02). The Wake anchor $H_{\textrm{opt}}^{\textrm{subj}}$ is therefore estimated from Wake epochs only, while the scale term $\Delta \alpha ^{\textrm{subj}}$ uses the empirical $\bar{\alpha }_{\textrm{dfa}}$ range across all stages for that subject. The mapping is analogous to z-scoring relative to a within-subject baseline where the location (anchor) is estimated from the reference condition and the spread is estimated empirically. We state this dependence explicitly because it is part of the domain-adaptation procedure and not a parameter tuned on the test contrast. The recalibration is methodologically necessary because synthetic high-consciousness states cluster at $\alpha _{\textrm{dfa}}\approx 0.28$–0.36 (Kuramoto simulator), whereas real waking EEG has $\alpha _{\textrm{dfa}}\approx 1.07$ (genuine 1/*f* dynamics). The range-normalisation is preferred for the real-EEG setting because it is scale-adaptive to the compressed empirical $\alpha _{\textrm{dfa}}$ distribution (range ≈0.18 across stages, against a synthetic range of ≈1.1). The tuned quantity $H_{\textrm{eff}}$ serves as the scale-free temporal organisation component.

### Organised cross-frequency complexity

Organised cross-frequency complexity *D* captures structured interactions between slow and fast oscillatory components and temporal signal richness. We focus on theta–gamma phase–amplitude coupling.

For each channel *i*, we band-pass filter in the theta (4–8 Hz) and gamma (30–**45** Hz for real EEG analyses to respect the Nyquist limit of 100 Hz recordings; 30–80 Hz for synthetic data) ranges to obtain $x^{\theta }_i(t)$ and $x^{\gamma }_i(t)$. Applying the Hilbert transform yields the instantaneous theta phase $\phi _i(t)$ and gamma amplitude $A_i(t)$. Collecting across channels gives vector processes $\phi (t)=(\phi _i(t))_{i=1}^C$ and $A(t)=(A_i(t))_{i=1}^C$.

Phase–amplitude coupling is quantified using the Tort Modulation Index (Tort et al. 2010), which measures the Kullback–Leibler divergence of the observed gamma-amplitude distribution across 36 theta-phase bins from a uniform reference distribution. An amplitude guard is then applied, i.e., if the mean gamma envelope (in z-scored units) is below 0.001, *D* is set to zero to prevent spurious phase-amplitude coupling (PAC) estimates from near-zero gamma content. This guard is essential for the seizure state, where the burst envelope would otherwise produce artefactual PAC. We emphasise that the quantity $I_{\phi A}$ entering the composite (Eq. 5) is the raw Tort Modulation Index, not a surrogate-normalised *z*-score. Time-shift surrogates (50 per epoch, lag *T*/4 to 3*T*/4) are computed separately as a diagnostic check on whether the observed PAC can be distinguished from phase-randomised null distributions (Aru et al. 2015). The resulting surrogate *z*-scores are reported as an interpretive aid and are not inputs to the composite. Time-shift surrogates reduce the risk of trivial phase–amplitude alignment, but they do not fully control for PAC inflation caused by non-sinusoidal waveform shape, sharp transients, or asymmetric oscillatory morphology. Surrogate *z*-scores are therefore treated as diagnostic rather than definitive evidence of physiological PAC. In the real EEG analyses (N=30 Sleep-EDF subjects, two-channel recordings), mean surrogate *z*-scores across all stages and subjects were -0.03 to 0.15, confirming that theta–gamma PAC is not reliably detectable in two-channel recordings. The raw Tort MI nevertheless carries subject-level rank information across stages, which is what the benchmarking exploits; we return to this distinction between absolute-scale detectability and rank-level discriminability in Section 6.9.

To incorporate temporal complexity, we compute the analytic amplitude envelope of a broadband (1–40 Hz) component for each channel, binarise it at the median, and estimate Lempel–Ziv complexity (Lempel and Ziv 1976). The binary sequence is normalised by $n/\log _2 n$ Höhn et al. (2024). Averaging across channels yields *LZ*.

Organised cross-frequency complexity is then,

5 $$\begin{aligned} D = I_{\phi A}\bigl (1+\lambda \,LZ\bigr ), \end{aligned}$$

where $I_{\phi A}$ is the Tort Modulation Index described above, and λ (λ=1.0) controls the contribution from the complexity term. This makes *D* sensitive to both cross-frequency coupling and temporal richness. The multiplicative form is a modelling choice rather than a derived law, and its single free parameter λ leaves the composite essentially unchanged across the range we examined. In the two-channel empirical setting analysed later, the broadband term varies little across states, so *D* behaves almost entirely as a rescaling of the raw theta–gamma index $I_{\phi A}$ (Section 6.9).

### Metastability

Metastability is quantified via the variability of phase synchronisation in the alpha band (8–13 Hz) Tognoli and Kelso (2014); Hancock et al. (2023). For each channel *i*, we extract the alpha-band component and compute its analytic phase $\theta _i(t)$. The Kuramoto order parameter at time *t* is,

6 $$\begin{aligned} R(t)=\left| \frac{1}{C}\sum _{i=1}^{C}\exp (i\theta _i(t))\right| . \end{aligned}$$

This quantity approaches 1 when phases are aligned and 0 when uniformly distributed. Metastability is the standard deviation of *R*(*t*) over time,

7 $$\begin{aligned} M=\textrm{std}_t\bigl (R(t)\bigr ). \end{aligned}$$

High metastability reflects frequent alternations between synchronised and desynchronised states. Note that, with only two EEG channels, the Kuramoto order parameter reduces to a pairwise phase-lag measure. Metastability claims here therefore apply to the 16-channel synthetic configuration, i.e., the two-channel real-EEG estimate of *M* is treated as an indicative synchrony measure only (see Section 6.9).

### Composite index

For each state, we compute $H_{\textrm{eff}}$, *D*, and *M*. We normalise each metric across states using min–max normalisation with respect to a reference ensemble. In the analyses reported here, this reference ensemble is the 270-run Monte Carlo ensemble comprising 30 runs per each of the nine simulated states. If $v^{(c)}$ denotes metric *v* for state *c*, the normalised quantity is defined as,

8 $$\begin{aligned} v_{\textrm{norm}}^{(c)}=\frac{v^{(c)}-\min _{c'}v^{(c')}}{\max _{c'}v^{(c')}-\min _{c'}v^{(c')}}. \end{aligned}$$

The composite index for state *c* is,

9 $$\begin{aligned} \Psi ^{(c)}=w_H H_{\textrm{eff,norm}}^{(c)}+w_D D_{\textrm{norm}}^{(c)}+w_M M_{\textrm{norm}}^{(c)}, \end{aligned}$$

where $w_H=0.40$, $w_D=0.35$, $w_M=0.25$ (chosen a priori, not optimised on evaluation data), with $w_H + w_D + w_M = 1$.

We note that pairwise Spearman correlations among the three components on the 270-run Monte Carlo ensemble are, $H_{\textrm{eff}}$–*D*, r=0.62; $H_{\textrm{eff}}$–*M*, r=0.61; *D*–*M*, r=0.34. These moderate correlations reflect the fact that all three components respond to the conscious/non-conscious manipulation. The components are therefore complementary rather than strictly independent, and in the two-channel empirical setting (Section 6.9) the discriminative signal is carried mainly by $H_{\textrm{eff}}$ together with the raw cross-frequency term, so the composite there does not exceed its strongest single component. Normalisation ensures scale compatibility across metrics, making the weighted sum a transparent and principled summary. Sensitivity analyses (Section 6.7) demonstrate that Ψ remains robust to moderate weight perturbations (±0.1).

**Fig. 1:**
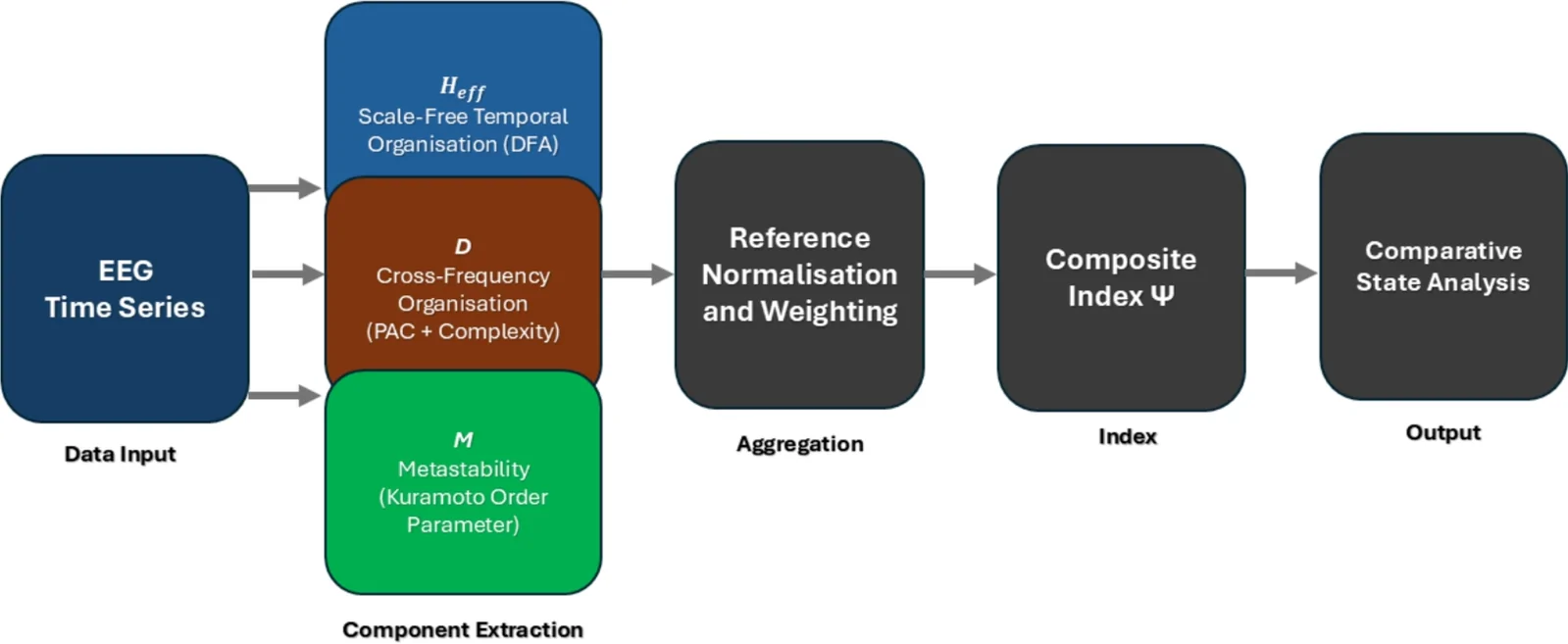
Analysis pipeline. EEG time series yield three components, $H_{\textrm{eff}}$ (scale-free temporal organisation), *D* (cross-frequency organisation), and *M* (metastability), which are reference-normalised and weighted to form the composite index Ψ

Figure 1 summarises the complete pipeline, from the three component estimates through reference normalisation and weighting to the composite index.

## Generative model and data generation

### Simulation of EEG-like signals

Here, we generate synthetic EEG-like signals. Each simulation produces *C* channels sampled at 250 Hz for 5–20 seconds, depending on robustness tests. All channels are z-scored (zero mean, unit variance) per channel before metric computation. Baseline activity per channel is generated by sampling white Gaussian noise, integrating to approximate a 1/*f* spectrum, and normalising to unit variance, yielding wide-band signals with scale-free-like fluctuations (He 2010).

We superimpose oscillatory components in five canonical bands such that delta (1–4 Hz), theta (4–8 Hz), alpha (8–13 Hz), beta (13–30 Hz), and gamma (30–80 Hz). For each band, we generate a global source via filtered independent noise and local components via filtered baseline per channel. A band-specific global mixing parameter controls the common oscillatory drive versus independent activity. Band components are scaled by state-specific weights, producing distinct spectral profiles.

Cross-channel interaction is introduced via a Kuramoto-like model applied to band-limited components. Phases evolve as,

10 $$\begin{aligned} \theta _i(t+1)=\theta _i(t)+\omega _i+K\sum _j A_{ij}\sin \bigl (\theta _j(t)-\theta _i(t)\bigr )+\xi _i(t), \end{aligned}$$

where $\omega _i$ are intrinsic frequencies, *K* is a coupling constant, $A_{ij}$ is a sparse connectivity matrix, and $\xi _i(t)$ is noise. This produces regimes from incoherent oscillations to strong synchrony.

Phase–amplitude coupling is implemented by modulating gamma-band amplitude via theta-band phase,

11 $$\begin{aligned} X_i(t)\leftarrow X_i(t)+\eta _{\textrm{PAC}}\sin (\theta _i^{\theta }(t))A_i^{\gamma }(t), \end{aligned}$$

where $\eta _{\textrm{PAC}}$ controls coupling strength, $\theta _i^{\theta }(t)$ is the theta phase, and $A_i^{\gamma }(t)$ is the gamma amplitude.

Seizure-like bursts are added as high-amplitude, narrow-band oscillations with Gaussian temporal envelopes to a large channel subset, with frequencies and timings randomly sampled within state-specific ranges (Jiruska et al. 2013). The amplitude guard (D=0 when the mean gamma envelope falls below 0.001 SD of the z-scored signal) is applied *after* per-channel z-scoring and *after* band-pass filtering to the 30–80 Hz gamma range (30–45 Hz for real EEG), operating on the Hilbert envelope of the filtered signal averaged across channels. The seizure state falls below this threshold because, although the raw seizure signal contains high-amplitude bursts, those bursts are narrow-band oscillations at low frequencies (0.5–4 Hz in the generative model); the 30–80 Hz gamma band therefore captures very little genuine power, and per-channel z-scoring rescales the broadband signal so that the narrow band-pass output has a small envelope magnitude relative to the 1 SD of the z-scored input. Without this guard, the Tort MI detects the burst envelope as spurious PAC (Monte Carlo mean D≈0.47, $\Psi \approx 0.38$); with it, Seizure D=0 and the Monte Carlo mean $\Psi \approx 0.10$—below Sleep ($\Psi \approx 0.49$)—consistent with the empirical literature (Jiruska et al. 2013). We note that ictal phenomenology is heterogeneous, i.e., focal aware seizures can preserve consciousness, and the dynamical signature modelled here corresponds specifically to hypersynchronous generalised seizure activity, not to seizures in general.

All signals are normalised post-simulation to minimise amplitude differences, ensuring metrics reflect structural rather than trivial amplitude variations.

The generative model is biologically plausible yet abstract. State-specific manipulations reflect empirical signatures, i.e., (i) psychedelics increase broadband desynchronisation, reduce low-frequency stability, and enhance CFC; (ii) NREM sleep and anaesthesia strengthen low-frequency synchrony and suppress organised CFC; (iii) REM dreaming restores high-frequency complexity with reduced low-frequency integration; (iv) seizures produce hypersynchronous, low-dimensional dynamics with minimal metastability.

### Simulated states

We simulate nine brain state classes. Psychedelic states feature increased gamma activity, enhanced theta–gamma coupling, and high metastability. Wakeful rest shows balanced band weights with prominent alpha/beta rhythms, moderate CFC, and strong metastability. Task-engaged states resemble wakeful rest but with stronger high-frequency power and more pronounced CFC, reflecting increased information processing.

Dreaming states retain substantial CFC and metastability but have altered band weights (reduced alpha, increased theta/low gamma), consistent with preserved complexity during REM sleep (Siclari et al. 2017; Sarasso et al. 2021; Tononi et al. 2024). NREM-like sleep states exhibit increased delta power, reduced high-frequency activity, weaker PAC, and lower metastability, aligning with breakdowns in effective connectivity and complexity (Massimini et al. 2005; Tagliazucchi et al. 2016). Minimally conscious states combine sleep and wakefulness elements, with modest CFC and intermediate metastability.

**Table 1:** Raw and derived metrics for single realisations of each simulated state. $\alpha _{\textrm{dfa}}$ is the mean DFA scaling exponent; *D*, *M*, and $H_{\textrm{eff}}$ are the three components; Ψ is the normalised composite. The tabulated $H_{\textrm{eff}}$ values are obtained by applying the Gaussian tuning function $H_{\textrm{eff}} = \exp [-(\bar{\alpha }_{\textrm{dfa}} - H_{\textrm{opt}})^2 / (2\sigma _H^2)]$ with $H_{\textrm{opt}} = 0.35$, $\sigma _H = 0.12$ to the tabulated $\bar{\alpha }_{\textrm{dfa}}$ (rounded to three decimal places). The composite Ψ uses min–max normalisation against the 270-run Monte Carlo ensemble bounds, not against within-table extremes, so Ψ cannot be reconstructed arithmetically from the single-realisation component values alone. Values are from a single representative simulation per state and therefore differ substantively from the 30-run Monte Carlo averages in Figure 3; the Monte Carlo means should be read as the primary quantitative result. The amplitude guard sets D=0 for Seizure, yielding Ψ=0.20 in this single realisation (below Sleep at 0.22); the corresponding Monte Carlo means are Seizure $\Psi \approx 0.10$ and Sleep $\Psi \approx 0.49$

State	$\alpha _{\textrm{dfa}}$	*D*	*M*	$H_{\textrm{eff}}$	Ψ
Psychedelic	0.340	0.032	0.187	0.997	0.73
Wake	0.386	0.027	0.242	0.956	0.91
Task-engaged	0.361	0.028	0.220	0.996	0.91
Dreaming	0.354	0.029	0.207	0.999	0.72
Sleep (NREM-like)	0.808	0.030	0.177	0.001	0.22
Minimally Conscious	0.815	0.028	0.171	0.001	0.22
Anaesthesia	0.807	0.029	0.153	0.001	0.18
Non-conscious	1.375	0.029	0.112	0.000	0.18
Seizure	1.364	0.000	0.001	0.000	0.20

Anaesthesia and non-conscious states show strong low-frequency global synchrony, suppressed high-frequency activity, minimal PAC, and reduced metastability, echoing propofol and other anaesthetics (Schartner et al. 2015; Bonhomme et al. 2019; Montupil et al. 2023; Casey et al. 2024). Seizure states are driven by high-amplitude, hypersynchronous bursts with stereotyped patterns and reduced metastability (Jiruska et al. 2013). Table 1 summarises single-realisation metrics after normalisation.

Table 2 reports the two state-specific parameters most directly relevant to the dynamical mechanisms examined here, namely the Kuramoto coupling constant *K*, which controls cross-channel phase interaction, and the PAC strength $\eta _{\textrm{PAC}}$, which controls theta–gamma phase–amplitude coupling. The remaining simulator settings, including band weights, noise scale, slow-drift amplitude, and seizure-burst parameters, are specified in full in the released code.

**Table 2:** State-specific generative parameters. *K* is the Kuramoto coupling constant governing cross-channel phase interaction and $\eta _{\textrm{PAC}}$ is the theta–gamma phase–amplitude coupling strength. Larger *K* yields stronger large-scale synchrony and larger $\eta _{\textrm{PAC}}$ yields stronger cross-frequency coupling. The non-conscious and seizure states use no network coupling, with seizure hypersynchrony arising instead from a shared global drive.

State	*K*	$\eta _{\textrm{PAC}}$
Psychedelic	0.70	0.70
Wake	0.60	0.40
Task-engaged	0.55	0.55
Dreaming	0.42	0.20
Sleep (NREM-like)	0.40	0.05
Minimally Conscious	0.32	0.060
Anaesthesia	0.10	0.005
Non-conscious	0.00	0.00
Seizure	0.00	0.00

## Results

Sections 6.1–6.8 demonstrate that the pipeline reliably recovers the dynamical motifs deliberately planted in the generative model, and characterise the composite’s robustness properties (ablation, hyperparameter sensitivity, Monte Carlo convergence). These synthetic analyses demonstrate internal consistency and the non-substitutability of each component, but do not by themselves constitute evidence that Ψ tracks phenomenal consciousness in biological data. Section 6.9 presents the independent empirical test using real Sleep-EDF EEG.

### Single-realisation components and composite index

**Fig. 2:**
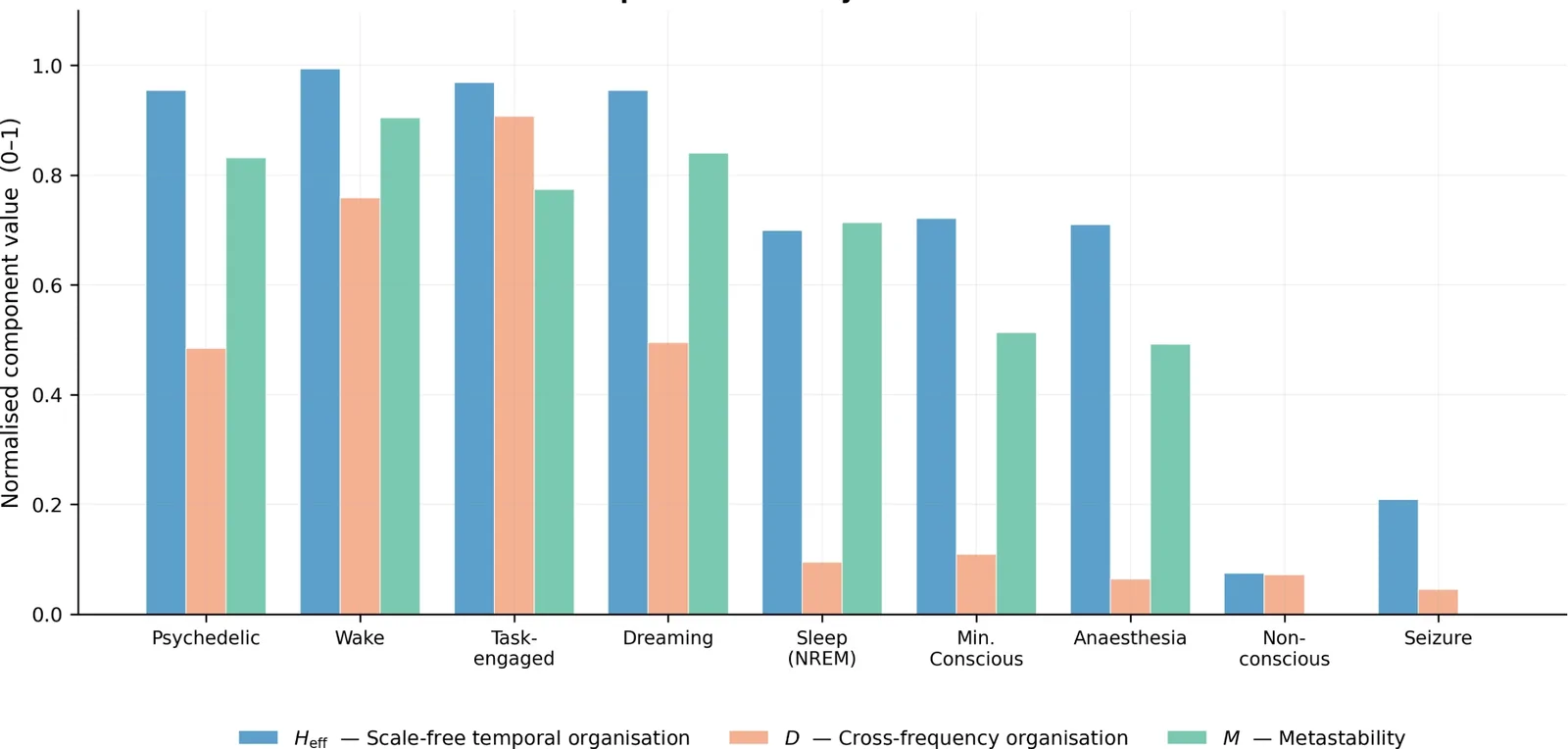
Normalised component values for $H_{\textrm{eff}}$, *D*, and *M* across simulated states. High-consciousness states show elevated values on all components; dreaming retains high $H_{\textrm{eff}}$ and *M* with moderate *D*; low-consciousness states show broadly suppressed components

**Fig. 3:**
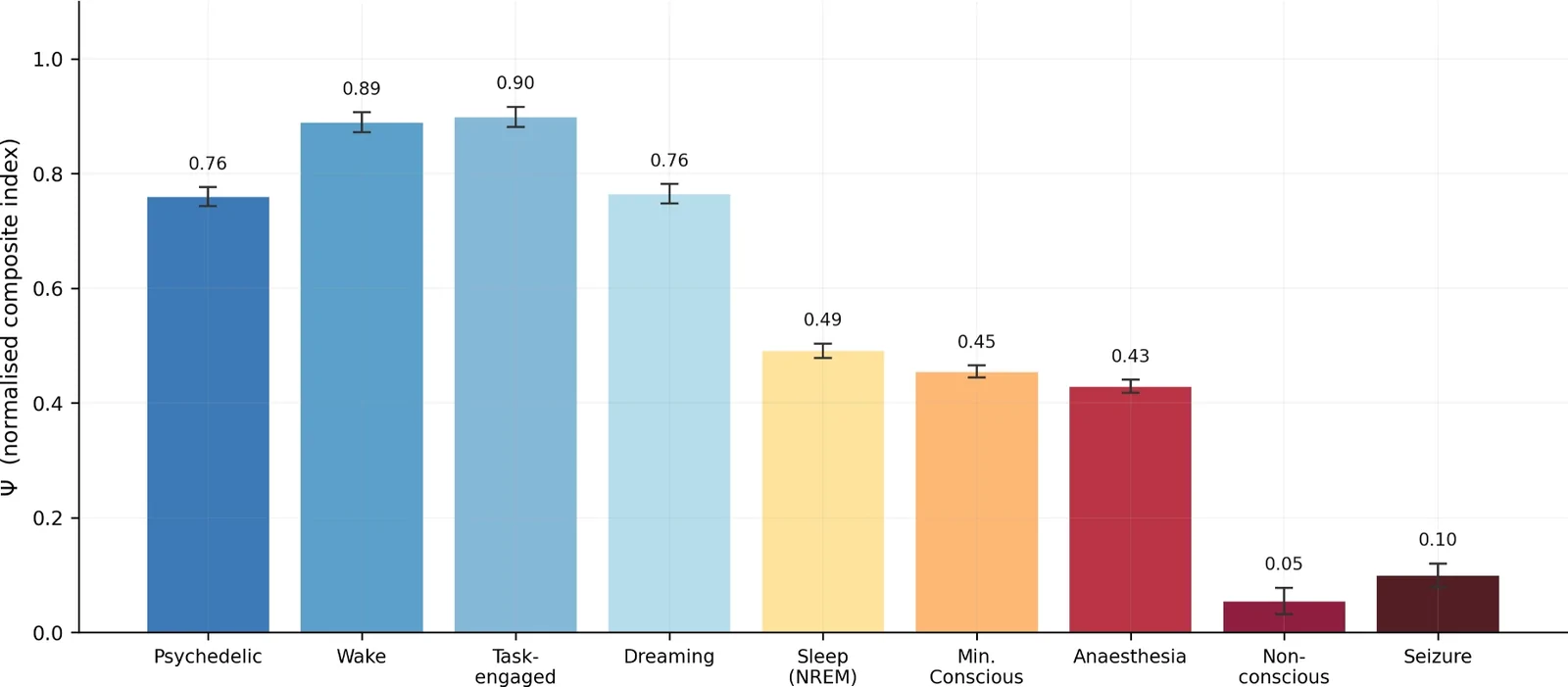
Composite index Ψ across the nine simulated brain states, computed as means over 30 Monte Carlo runs per state (error bars: ± SD). High-consciousness states cluster at 0.76–0.90; low-vigilance states at 0.43–0.49; non-conscious and seizure states at 0.05–0.10. The Seizure result follows amplitude-guard correction, which prevents burst-envelope artefacts from producing spurious PAC

Table 1 reports DFA scaling exponents ($\alpha _{\textrm{dfa}}$), cross-frequency complexity, metastability, tuned scale-free temporal organisation $H_{\textrm{eff}}$, and composite index Ψ for single realisations of each state. Figure 2 shows normalised component values $H_{\textrm{eff,norm}}$, $D_{\textrm{norm}}$, and $M_{\textrm{norm}}$ as a grouped bar chart. Figure 3 displays the composite index Ψ per state.

The component plot shows that wake-like and high-consciousness synthetic states have the highest scale-free temporal organisation and metastability, with substantial cross-frequency complexity. Psychedelic states show slightly reduced scale-free temporal organisation relative to wake, consistent with the “entropic brain” hypothesis (Carhart-Harris et al. 2014; Montupil et al. 2023), but maintain high CFC and metastability, placing them in the same high-consciousness cluster as wakefulness, although the Monte Carlo mean Ψ for Psychedelic (≈0.76) is below that of Wake (≈0.89). Dreaming exhibits high organised complexity and metastability but moderate scale-free temporal organisation, aligning with preserved complexity and connectivity during REM sleep (Siclari et al. 2017; Sarasso et al. 2021; Tononi et al. 2024). Seizure states show D=0 (amplitude guard removes burst-envelope artefact) and low metastability, yielding a Monte Carlo mean $\Psi \approx 0.10$ (see Figure 3), well below the Sleep Monte Carlo mean of $\Psi \approx 0.49$, consistent with hypersynchronous, low-dimensional dynamics (Jiruska et al. 2013). Anaesthesia and non-conscious states have low values across components, especially scale-free temporal organisation and CFC.

### Monte carlo distributions across states

**Fig. 4:**
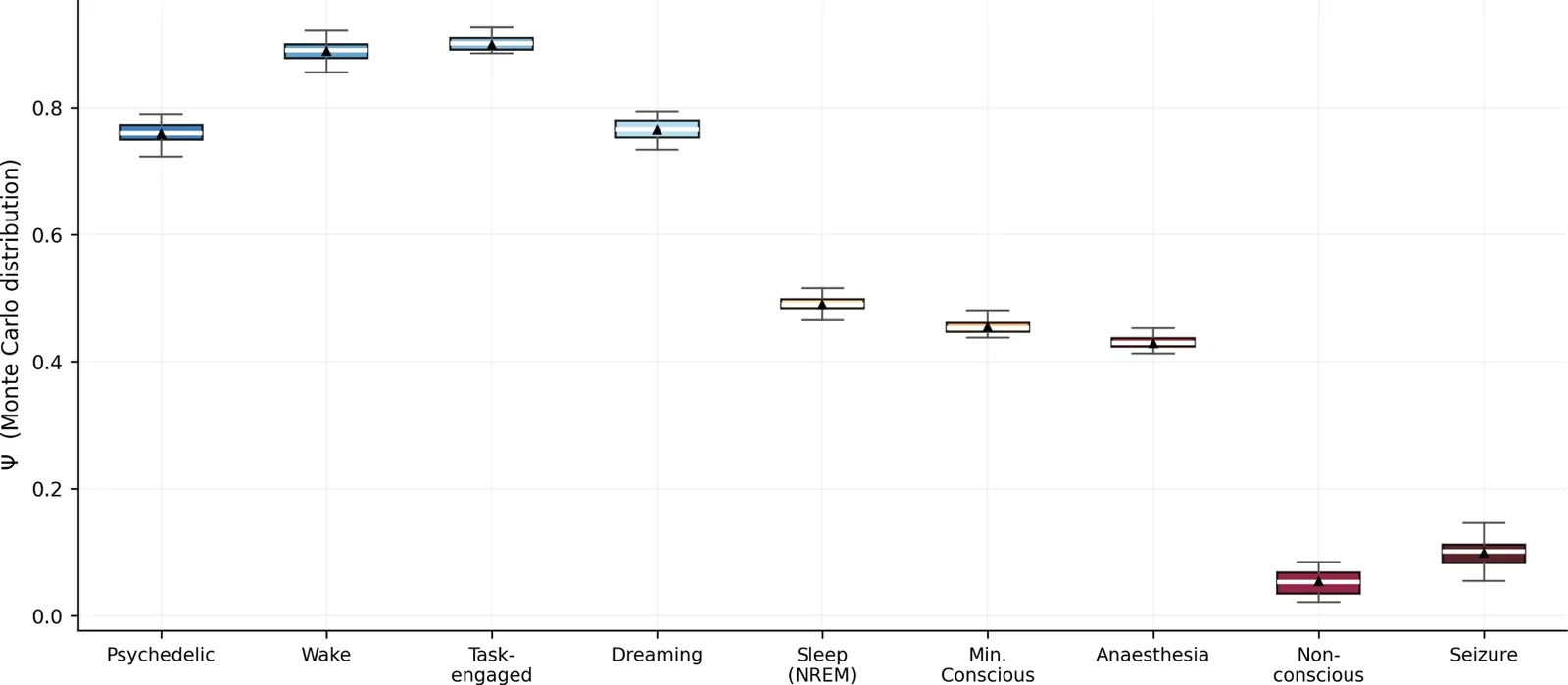
Monte Carlo distribution of Ψ across simulated states, based on 30 runs per state. High-consciousness states cluster at the upper end, intermediate states in the middle, and low-consciousness or non-conscious states at the lower end

To assess stability under stochastic variability, we generated thirty independent simulations per state, recomputed all metrics, and examined the composite index distribution. Figure 4 shows box-and-whisker plots of the Monte Carlo composite index $\Psi _{\textrm{mc}}$ across states.

Psychedelic, wake, and task-engaged states maintain high median $\Psi _{\textrm{mc}}$ values (0.76–0.90) with modest spread. Dreaming states cluster slightly lower but clearly above non-conscious and anaesthetic conditions. Non-rapid eye movement sleep and minimally conscious states occupy the mid-lower region with overlapping but distinct distributions. Non-conscious and Seizure states remain tightly clustered near the bottom ($\Psi \approx 0.05$–0.10). The across-state Kruskal–Wallis omnibus is highly significant (H=260.42, $p = 1.06 \times 10^{-51}$), and all eight adjacent state transitions separate with large rank-based effect sizes (Cliff’s $\delta \ge 0.83$ for all but one pair).

### Conscious versus non-conscious classification

**Fig. 5:**
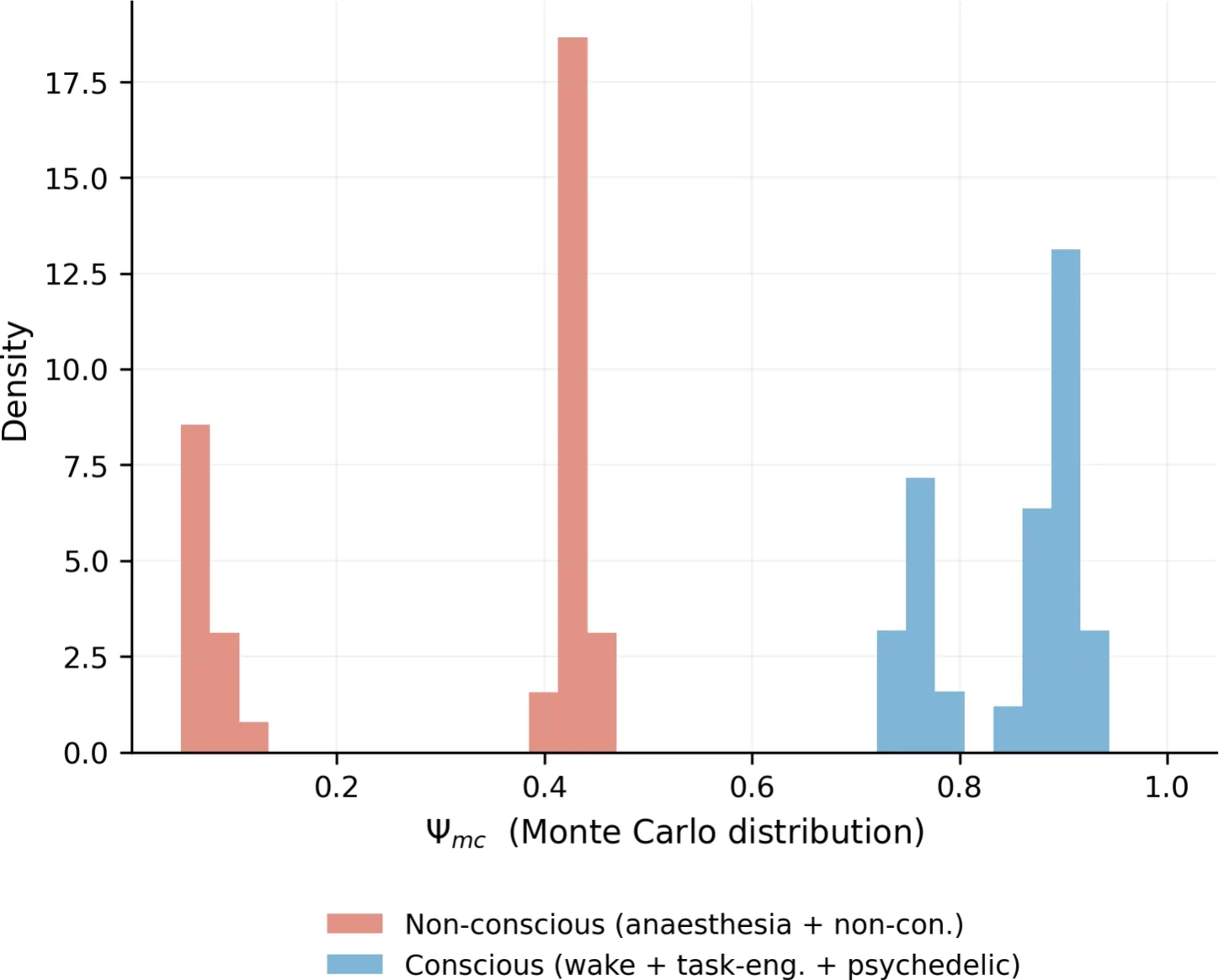
Kernel-smoothed histograms of Ψ for conscious (wake + task-engaged + psychedelic; blue) and non-conscious (anaesthesia + non-conscious; orange) Monte Carlo runs. The distributions are non-overlapping

We grouped psychedelic, wake, and task-engaged states into a “conscious” class and anaesthesia and non-conscious states into a “non-conscious” class, pooling Monte Carlo runs. We retain the “conscious” and “non-conscious” labels here as pooled-class identifiers for the binary classifier analysis only; they are not phenomenological claims about individual states. Dreaming, for example, is a conscious state that we deliberately exclude from this pooling to keep the classifier contrast unambiguous, not a denial that REM dreaming is conscious. Sleep, minimally conscious, and seizure states are also excluded from the pooled contrast because they are intermediate or pathological. Sleep and minimally conscious states occupy the middle of the Ψ range and do not cleanly sit on either side of a binary conscious/non-conscious split, and seizure is excluded because the generative model targets a specific hypersynchronous dynamical signature that is not representative of unconsciousness in general. Figure 5 displays kernel-smoothed histograms of $\Psi _{\textrm{mc}}$ for both classes.

The non-overlapping distributions indicate that, within this synthetic environment, Ψ captures the dynamical organisation differences built into the generative model. Quantitatively, the conscious-versus-non-conscious classification achieves AUC =1.000 with a 95% bootstrap confidence interval of [1.000, 1.000] ($n_{\textrm{conscious}} = 90$ runs, $n_{\mathrm {non-conscious}} = 60$ runs), i.e., the distributions are strictly non-overlapping across the 150 Monte Carlo realisations contributing to these two classes.

### Robustness to duration, channel count and noise

We assessed robustness by systematically varying recording duration (5, 10, 20 seconds), channel count (8, 16, 32), and additive Gaussian noise level (standard deviations 0, 0.3, 0.6) for wake and anaesthesia states. Robustness analyses were also conducted for dreaming and minimally conscious states. For each configuration, $\Psi _{\textrm{mc}}$ was computed across thirty realisations, and the standard deviation was estimated across the combined ensembles.

Increasing duration systematically reduced variability, consistent with improved averaging from longer time series. Increasing channel count modestly reduced variability, reflecting stabilisation from larger spatial samples. Low-to-moderate noise caused only slight increases in variability, whereas higher noise levels increased variability more substantially as noise began to dominate the signal structure. Nevertheless, wake–anaesthesia distinctions remained evident even under high noise, though with reduced effect size.

These findings indicate graceful degradation under common variability sources. Robustness to realistic noise, duration, and montage variations supports potential empirical and clinical applicability, complementing evidence that complexity-based metrics can remain informative under challenging conditions (Sarasso et al. 2021; Casey et al. 2024; Liu et al. 2023).

### Component discriminability in synthetic data

**Fig. 6:**
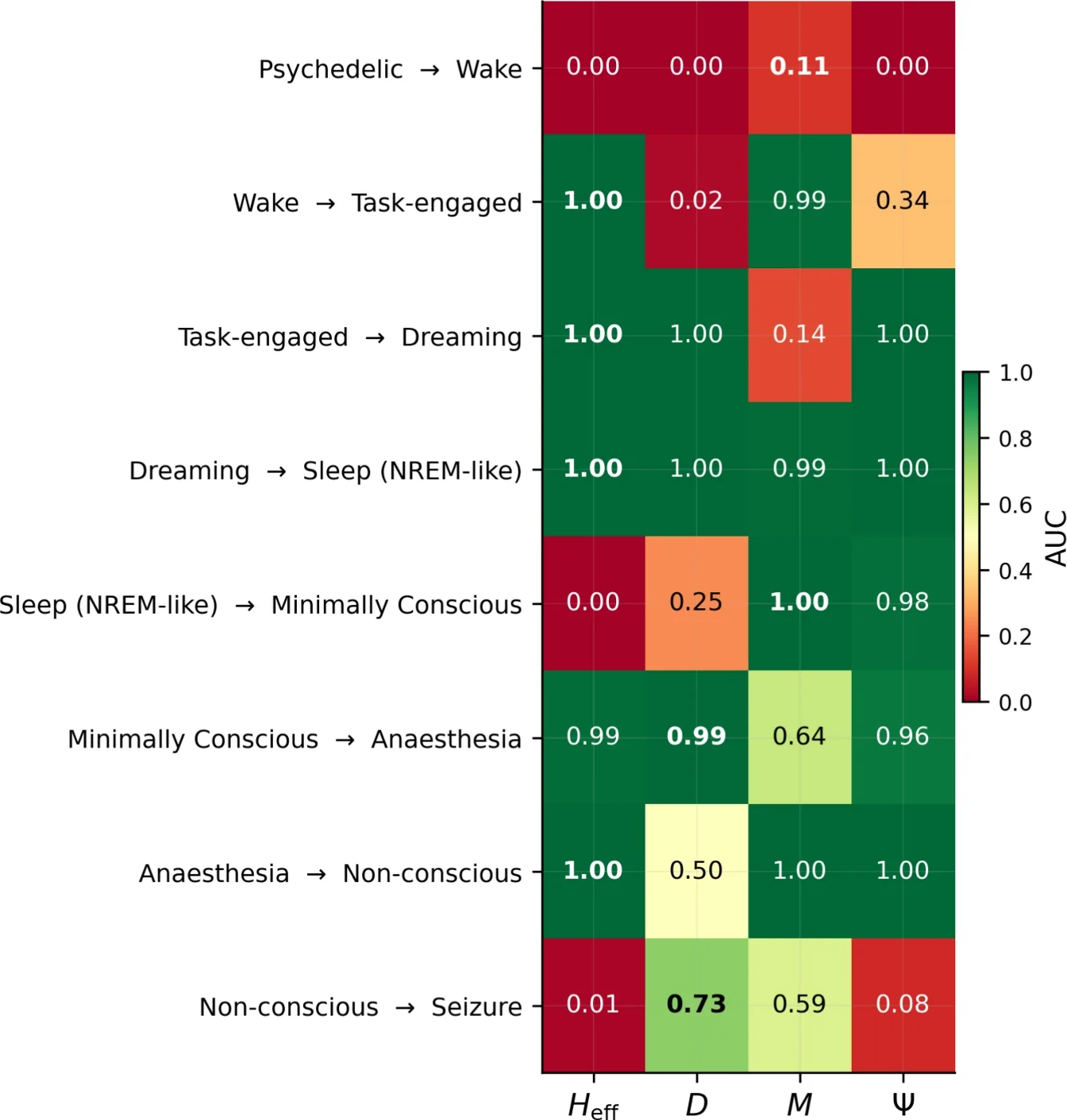
Pairwise AUC heatmap for adjacent synthetic state transitions (270-run Monte Carlo ensemble). Bold values mark the best component per row. Green cells indicate high AUC; red cells indicate AUC below 0.5 (including the two design-inverted transitions, Psychedelic → Wake and Non-conscious → Seizure). The Sleep → Minimally Conscious row illustrates the rescue mechanism of the composite, i.e., $H_{\textrm{eff}}$ alone fails (AUC =0.00), *M* recovers the discrimination (AUC =1.00), and Ψ inherits the correct ordering (AUC =0.98)

To evaluate the contribution of each component and the composite index to pairwise state discrimination, we computed AUC values for all eight adjacent state-pair transitions using the 270-run Monte Carlo ensemble. Figure 6 shows the results as a heatmap. No single component achieves the best discrimination on every transition. Four transitions are best discriminated by $H_{\textrm{eff}}$ (Wake → Task-engaged, Task-engaged → Dreaming, Dreaming → Sleep, Anaesthesia → Non-conscious), two by *D* (Minimally Conscious → Anaesthesia and Non-conscious → Seizure), and two by *M* (Psychedelic → Wake and Sleep → Minimally Conscious). The most striking rescue case is Sleep → Minimally Conscious, i.e., here $H_{\textrm{eff}}$ alone actively anti-discriminates (AUC =0.00) because the two states have similar DFA scaling, but *M* separates them perfectly (AUC =1.00), and the composite Ψ recovers a near-perfect 0.98. The Non-conscious → Seizure transition is design-inverted (Seizure Ψ> Non-conscious Ψ, reflecting the amplitude guard that sets Seizure D=0); here *D* is the best-discriminating component (AUC =0.73), with $H_{\textrm{eff}}$ actively anti-discriminating and *M* only marginally informative. These patterns establish that each of the three components contributes unique discriminative information somewhere along the state hierarchy, and that the composite Ψ rescues transitions that any single component would miss.

### Ablation study

To evaluate the individual contributions of specific dynamical mechanisms to the composite index Ψ, we conducted a systematic ablation analysis. Beginning with the complete “wake-like” model as our baseline, we generated three modified versions where we selectively suppressed one core component at a time, i.e., (i) cross-frequency phase–amplitude coupling (PAC), (ii) network-driven metastability, and (iii) fractal scale-free dynamics. When we eliminated PAC by setting $\eta _{\textrm{PAC}} = 0$, organised theta–gamma coupling was abolished, resulting in a substantial reduction in the complexity measure *D* and producing a marked decrease in Ψ, thereby highlighting the fundamental importance of cross-frequency coordination in conscious-like states. Suppressing metastability through reducing the Kuramoto coupling constant to $K = 10^{-3}$ eliminated coherent phase fluctuations, significantly diminishing metastability measure *M* while preserving $H_{\textrm{eff}}$ and *D* relatively unchanged. Removing fractal dynamics by fixing all DFA scaling exponents to $\alpha _{\textrm{dfa}}=0.5$ selectively reduced the scale-free temporal organisation score $H_{\textrm{eff}}$ with minimal impact on the other components.

**Fig. 7:**
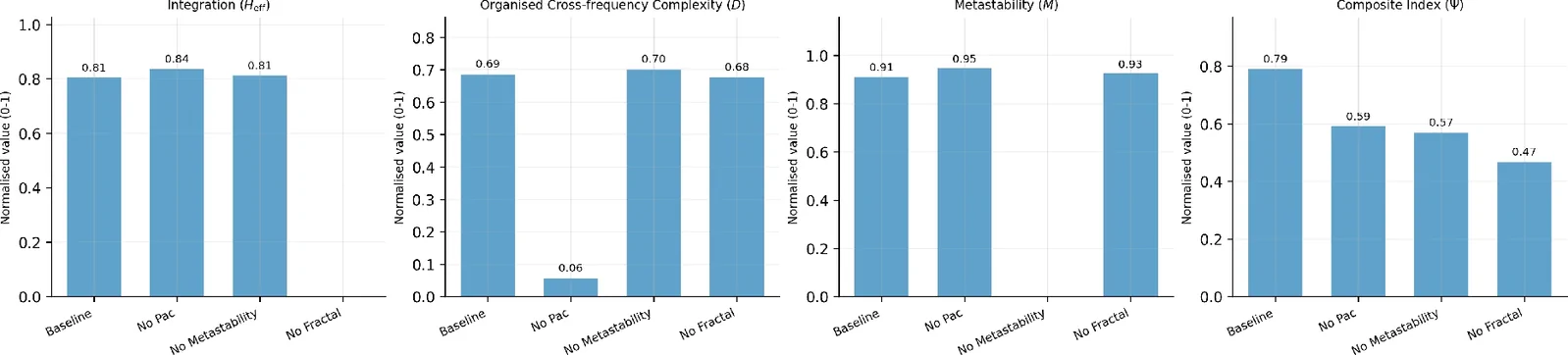
Ablation analysis. Each panel shows the effect of removing one component on all three components and on the composite. Targeted ablations selectively suppress their intended component (*D* collapses when PAC is removed; *M* collapses when metastability is suppressed; $H_{\textrm{eff}}$ collapses when fractal scaling is removed) while leaving others largely intact. The composite Ψ falls from a baseline of 0.79 to 0.59, 0.57, and 0.47, respectively, confirming that each component is non-redundant

Figure 7 quantifies the result of each ablation. The baseline composite Ψ=0.79 drops to 0.59 when PAC is removed (a reduction of 0.20), to 0.57 when metastability is removed (0.22), and to 0.47 when scale-free dynamics are removed (0.32). Each ablation produces a large, directly interpretable drop, confirming that no single component is redundant. Crucially, each ablation selectively affects its targeted component. For example, removing PAC collapses *D* (from 0.69 to 0.06) while $H_{\textrm{eff}}$ and *M* are essentially unchanged, and removing metastability drives *M* to zero while leaving $H_{\textrm{eff}}$ and *D* nearly identical. This pattern rules out collinearity as an explanation for the composite’s performance, and it establishes that each component is non-substitutable in the synthetic setting.

### Sensitivity analysis

**Fig. 8:**
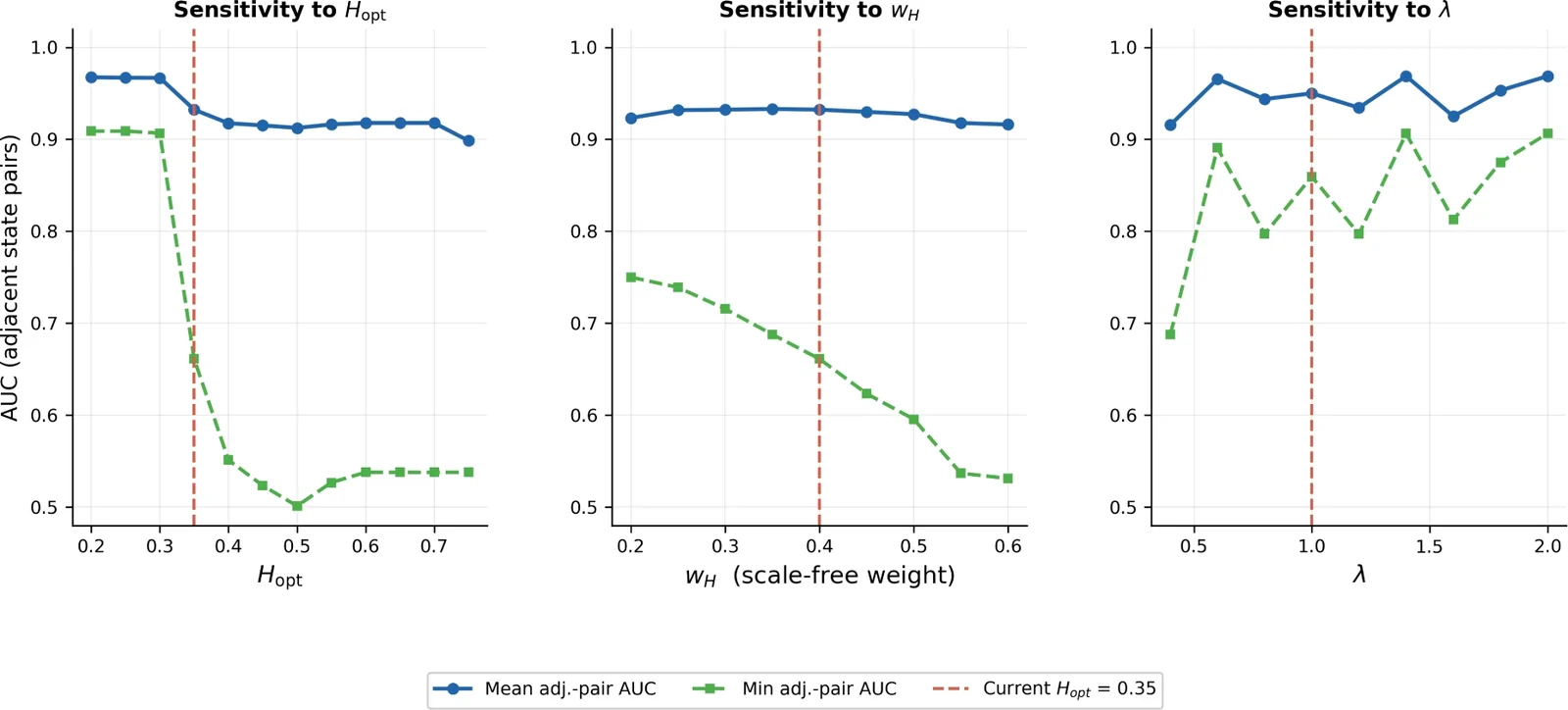
Hyperparameter sensitivity. Each panel sweeps one hyperparameter ($H_{\textrm{opt}}$, $w_H$, λ) while holding the others fixed. Solid blue marks the mean AUC across adjacent state pairs. Dashed green marks the worst pair AUC. Red dashed lines mark the chosen values ($H_{\textrm{opt}} = 0.35$, $w_H = 0.40$, λ=1.0). The chosen values for $H_{\textrm{opt}}$ and $w_H$ sit at the upper edge of their stable regions rather than at central plateaus; λ is chosen within a broad stable region

To confirm that the composite index Ψ is not over-fitted to the specific values of its hyperparameters, we conducted a systematic sensitivity analysis. For each hyperparameter ($H_{\textrm{opt}}$, $w_H$, and λ), we swept a wide range of values, recomputed Ψ on the 270-run Monte Carlo ensemble, and tracked two summary statistics, i.e., the mean AUC across all adjacent-state pairs (a measure of overall discrimination) and the minimum AUC across pairs (a measure of the weakest-link discrimination). Figure 8 shows the results.

The mean AUC (solid blue) remains in the range 0.90–0.97 across every hyperparameter sweep, confirming that the composite’s overall discrimination is robust. The minimum AUC (dashed green) is a more stringent test. It reveals the discrimination of the single worst-performing state pair at each parameter value. For $H_{\textrm{opt}}$, the minimum AUC holds near 0.90 for values in [0.20, 0.35] and drops sharply above 0.35; the chosen value $H_{\textrm{opt}} = 0.35$ (red dashed) therefore sits at the *upper edge* of the stable region. Values in [0.20, 0.35] preserve strong worst-pair discrimination, whereas values above 0.35 produce rapid degradation. The choice of 0.35 is conservative under the assumption that real EEG in an awake cohort typically exhibits higher $\bar{\alpha }_{\textrm{dfa}}$ than the synthetic regime, so values below 0.35 were preferred. An analogous pattern obtains for $w_H$. The minimum AUC degrades monotonically as $w_H$ decreases below 0.40, and the chosen value $w_H = 0.40$ lies at the upper shoulder. The λ panel shows that Ψ is largely insensitive to this hyperparameter (minimum AUC >0.68 across the entire [0.4, 2.0] range), with the chosen value λ=1.0 producing minimum AUC ≈0.86.

Collectively, these sweeps demonstrate that the framework was not tuned to a sharply-peaked optimum; the chosen values occupy broad stable regions of the hyperparameter space.

### Monte carlo convergence

**Fig. 9:**
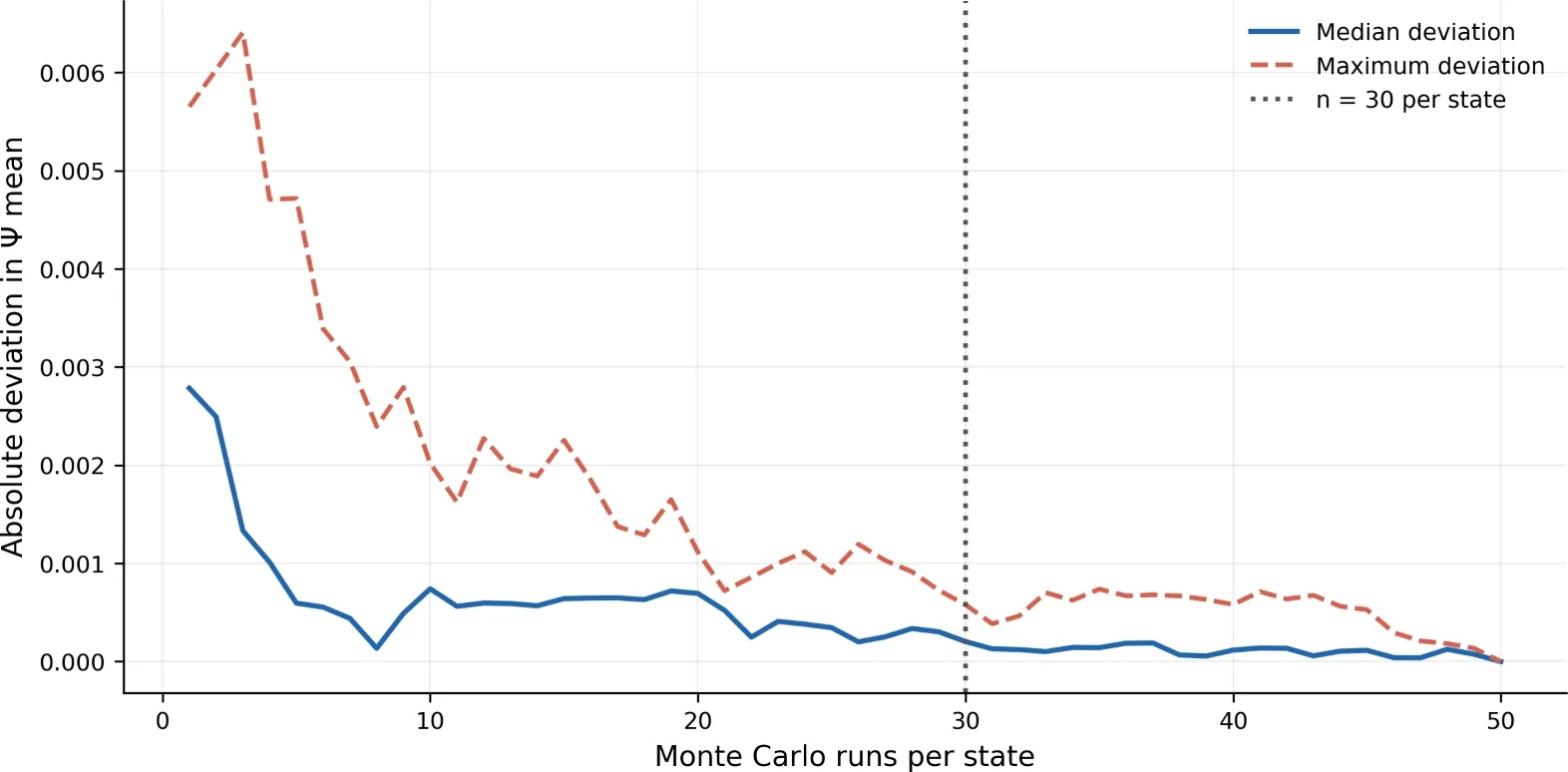
Monte Carlo convergence of the per-state Ψ mean towards its 50-run asymptotic value, as a function of runs per state. Solid blue marks median deviation across the nine states. Dashed red marks maximum deviation. At n=30 (dotted grey), both are below 0.001, confirming that the chosen sample size gives Ψ means precise to better than three decimal places

To justify the choice of 30 Monte Carlo runs per state, we examined the convergence of per-state Ψ means as the number of runs increases. Figure 9 plots the absolute deviation of the running-mean Ψ from the 50-run asymptotic value, summarised as the median and maximum across the nine states.

By n=20 runs per state, the maximum deviation across states is below 0.0015 and the median deviation is below 0.0008. At the chosen sample size n=30, both deviations are below 0.001, meaning that the reported per-state Ψ means are precise to better than three decimal places. This precision is well below the smallest effect size tested in the paper (the Wake vs. Task-engaged contrast, $\Delta \Psi = 0.01$) and well below the pairwise differences between adjacent-state categories ($\Delta \Psi \ge 0.28$ for the Sleep–Dreaming boundary). Thirty runs per state, therefore, provide adequate numerical precision for all discrimination claims in this paper.

### Validation of Ψ using real EEG

To assess the biological interpretability and robustness of Ψ, we conducted a validation study on real EEG from the Sleep-EDF Expanded dataset (PhysioNet). N=30 subjects (Sleep Cassette SC4001–SC4151) were included, yielding 1, 078 artefact-free 30-second epochs across Wake (W), N2 sleep, and REM sleep. Artefact rejection excluded epochs with peak amplitude exceeding $500\,\mu \textrm{V}$. Two EEG channels (Fpz-Cz and Pz-Oz) were used at their native 100 Hz sampling rate, with no upsampling. The gamma band was capped at 45 Hz for all real-EEG analyses (5 Hz guard band below Nyquist). Stratified epoch sampling distributed the ≤15 epochs per stage per subject evenly across the night to avoid confounding between time-of-night and stage. All signals were z-scored per channel before metric computation, matching the synthetic pipeline.

The unit of analysis is the subject-level mean Ψ per stage (computed by averaging across all artefact-free epochs for that subject in that stage). All statistical tests are paired within subjects. We used a Friedman omnibus test followed by pairwise Wilcoxon signed-rank tests with Bonferroni correction (threshold p<0.017), with matched-pairs rank-biserial correlation as the effect size. AUC values for all benchmarking comparisons were computed on subject-level means with 95% bootstrap confidence intervals (2,000 resamples over subjects).

**Fig. 10:**
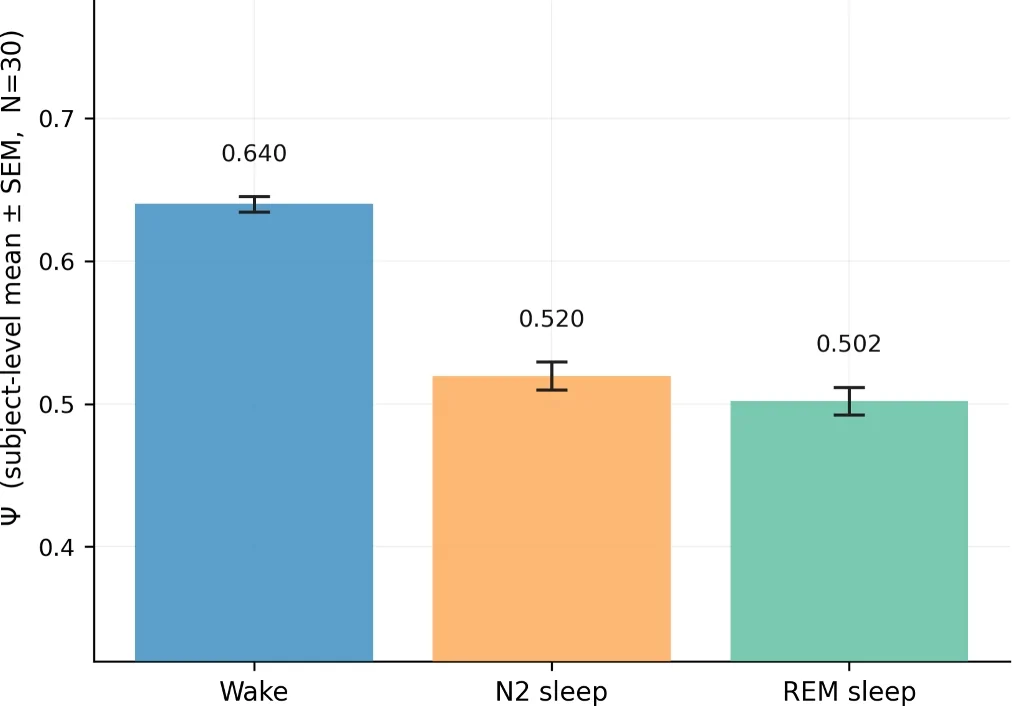
Subject-level mean Ψ (± SEM, N=30) for Wake, N2, and REM on Sleep-EDF (1, 078 epochs). Friedman omnibus $\chi ^2 = 45.27$, p<0.0001. Wake Ψ is significantly higher than both N2 and REM after Bonferroni correction (p<0.0001 each; matched-pairs rank-biserial $r_{rb} = +1.00$)

Figure 10 shows subject-level means ± SEM. The Friedman omnibus is highly significant ($\chi ^2 = 45.27$, p<0.0001, n=30 complete-case subjects, K=3 stages). Pairwise Wilcoxon tests show that Wake differs from both sleep stages at p<0.0001 (Bonferroni-significant) with matched-pairs rank-biserial $r_{rb} = +1.00$ for both contrasts—every one of the 30 subjects individually ranks Wake higher than N2, and every one ranks Wake higher than REM. The N2 versus REM contrast shows a medium effect size ($r_{rb} = +0.40$) that does not reach Bonferroni significance (p=0.058). Subject-level means (mean ± SEM) are, Wake =0.640±0.005 (SD =0.029); N2 =0.520±0.010 (SD =0.054); REM =0.502±0.010 (SD =0.052). The between-subject standard deviation is notably smaller in Wake than in sleep stages, indicating that Ψ during wakefulness is more consistent across individuals than during sleep.

**Fig. 11:**
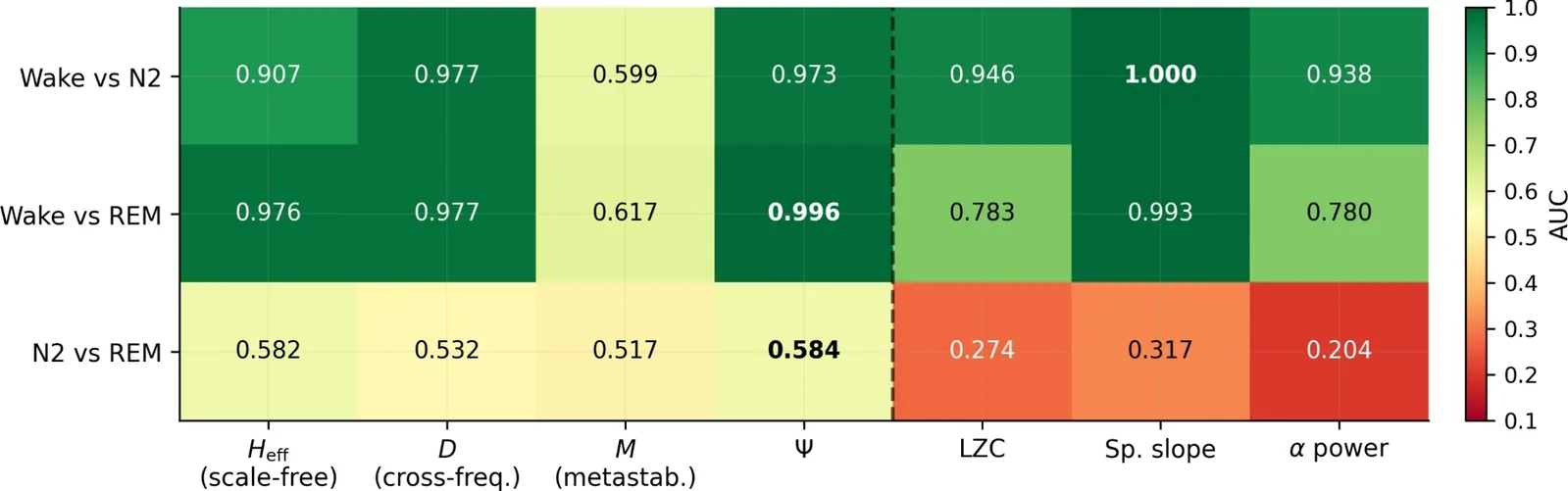
Empirical benchmarking heatmap. Subject-level AUC for the three components and the composite (left of dashed line) against three established markers—LZC, spectral slope, alpha power (right of dashed line)—on the N=30 Sleep-EDF data. Green marks AUC above chance; red marks AUC below chance. On N2 vs. REM, the three external baselines reverse the vigilance ordering (AUC ≤0.32), while the framework components and the composite Ψ lie close to chance. Ψ is nominally above 0.5, but its confidence-interval lower bound is close to chance. Bold values mark best performer per row

To benchmark Ψ against established single-metric markers, we computed subject-level AUC with 95% bootstrap confidence intervals for $H_{\textrm{eff}}$, *D*, *M*, Ψ, Lempel–Ziv complexity (LZC) (Höhn et al. 2024), spectral slope (Lendner et al. 2020; Brake et al. 2024), and alpha power on the same 1, 078 epochs. Table 3 and Figure 11 summarise the results.

Wake versus N2 is well-discriminated by multiple measures. Spectral slope achieves AUC =1.000 [1.000, 1.000], *D* and Ψ achieve 0.977 and 0.973 respectively, and LZC and alpha power lie in the 0.94 range. This is the expected result for a contrast between two states with distinct spectral signatures.

For wake versus REM, Ψ is the top-performing metric. Ψ AUC =0.996 [0.984, 1.000], narrowly ahead of spectral slope (0.993 [0.977, 1.000]) and well ahead of LZC (0.783) and alpha power (0.780). Both tested single-component metrics, $H_{\textrm{eff}}$ and *D*, also perform well (0.976 and 0.977), indicating that multiple components of the framework contribute a useful signal for this contrast.

The N2 versus REM contrast provides the most theoretically informative finding. All three established complexity baselines rank REM above N2 (LZC AUC =0.274 [0.211, 0.320], spectral slope AUC =0.317 [0.233, 0.376], and alpha power AUC =0.204 [0.120, 0.282]), contrary to the conventional vigilance ordering of N2 above REM. This direction is not a failure of the baselines, because the N2-versus-REM comparison is itself ambiguous for consciousness. REM supports vivid phenomenal experience and is by some measures the more wake-like of the two sleep stages, so a phenomenal-richness reading places REM above N2 whereas a vigilance reading places N2 above REM. The composite reaches AUC =0.584 [0.501, 0.676] and its components remain near chance ($H_{\textrm{eff}}$ =0.582, *D* =0.532, *M* =0.517), so none provides reliable separation between the two stages. The nominal N2-above-REM direction of Ψ follows from its subject-level Wake-anchored calibration rather than from any resolved discrimination, and with a confidence-interval lower bound at 0.501 and a paired Wilcoxon test at p=0.058 we take no position on which ordering is correct.

**Table 3:** Subject-level pairwise AUC with 95% bootstrap confidence intervals on Sleep-EDF (N=30; 2, 000 resamples). Bold marks best per row. Values below 0.5 indicate active anti-discrimination.

Stage pair	$H_{\textrm{eff}}$	*D*	*M*	Ψ	LZC	Sp. slope	α power
Wake vs. N2	0.907	0.977	0.599	0.973	0.946	$\mathbf {1.000}$	0.938
*95% CI*	[0.792, 0.986]	[0.940, 1.000]	[0.462, 0.743]	[0.933, 1.000]	[0.902, 0.983]	[1.000, 1.000]	[0.882, 0.981]
Wake vs. REM	0.976	0.977	0.617	$\mathbf {0.996}$	0.783	0.993	0.780
*95% CI*	[0.928, 0.999]	[0.930, 1.000]	[0.509, 0.734]	[0.984, 1.000]	[0.717, 0.860]	[0.977, 1.000]	[0.682, 0.871]
N2 vs. REM	0.582	0.532	0.517	$\mathbf {0.584}$	0.274	0.317	0.204
*95% CI*	[0.496, 0.671]	[0.436, 0.623]	[0.431, 0.600]	[0.501, 0.676]	[0.211, 0.320]	[0.233, 0.376]	[0.120, 0.282]

Finally, we examined the fractional contribution of each component to Ψ in real EEG. Per stage, the decomposition is Wake (Ψ=0.64), $H_{\textrm{eff}}$ contributes 51%, *D* contributes 10%, *M* contributes 39%; N2 (Ψ=0.52), $H_{\textrm{eff}}$ 48%, *D* 4%, *M* 48%; REM (Ψ=0.50), $H_{\textrm{eff}}$ 46%, *D* 4%, *M* 50%. No single component contributes more than 51% of Ψ’s value, meaning the composite does not collapse to any one component numerically. However, we note a distinction between *value contribution* and *discriminative contribution*. Although *M* accounts for roughly 39–50% of Ψ’s value across stages, its discriminative AUC for Wake-versus-sleep contrasts is modest (0.599–0.617) with wide confidence intervals. In the two-channel setting, *M* reduces to a pairwise phase-lag measure, limiting its ability to capture large-scale metastable dynamics. Similarly, *D* contributes 4–10% to Ψ but achieves AUC ≈0.98 for Wake-versus-sleep contrasts, demonstrating that subject-level rank information can be substantial even when the absolute scale of raw *D* is small. A direct decomposition clarifies what drives this. For the Wake-versus-sleep contrasts, the raw theta–gamma index $I_{\phi A}$ alone attains AUC 0.976 and 0.978, within rounding of *D* itself, while the broadband complexity term alone attains only 0.944 and 0.784. A dominance analysis over $I_{\phi A}$, the complexity term, and their product attributes the discrimination almost entirely to $I_{\phi A}$, with the multiplicative product contributing under one percent. The raw $I_{\phi A}$ is small in absolute terms, with subject-level means of about 0.0043 in Wake against 0.0020 in N2 and 0.0019 in REM, so its discriminative power reflects between-subject and between-stage rank rather than the absolute magnitude of theta–gamma coupling. Within subjects *D* and broadband complexity correlate only moderately (median Spearman ρ=0.41), so at two channels *D* acts as a between-subject ranking of raw theta–gamma modulation rather than as organised cross-frequency complexity in the intended sense. Multi-channel validation is required to engage *M* and *D* fully; see Section 7 for discussion.

The per-subject Wake-referenced calibration of $H_{\textrm{opt}}^{\textrm{subj}}$ warrants a brief note on robustness. Because the paired Wake-versus-sleep tests are rank-based, any monotonic rescaling of already-computed subject-level values leaves the matched-pairs rank-biserial statistic unchanged. Any calibration choice that preserves the per-subject ordering of Wake, N2, and REM $\bar{\alpha }_{\textrm{dfa}}$ yields the same $r_{rb}$. This post-hoc rank invariance does not by itself remove the need to report the calibration formula transparently. The scale term $\Delta \alpha ^{\textrm{subj}}$ uses the empirical range of $\bar{\alpha }_{\textrm{dfa}}$ across all retained stages for each subject, so the calibration is not entirely Wake-only. Cross-cohort calibration with a fixed $H_{\textrm{opt}}$ is a natural future direction.

## Discussion

We state the scope of our claim at the outset. The analyses do not establish that Ψ measures consciousness in any direct phenomenal sense, and the Sleep-EDF results should not be read as broad conscious-versus-unconscious discrimination. What the analyses at this stage support is that a composite of scale-free temporal organisation, cross-frequency organisation, and metastability behaves plausibly across nine synthetic state simulations and, on two-channel Sleep-EDF data, it separates wakefulness from sleep at the subject level, while neither the composite nor the established baselines reliably separate N2 from REM, and we make no claim about that within-sleep direction.

The synthetic results recover the qualitative state ordering expected from the consciousness-dynamics literature. High-consciousness states (wakefulness, task-engaged, psychedelic) cluster at the top, intermediate states (dreaming, NREM sleep, minimally conscious) occupy the middle range, and non-conscious, anaesthetised, and seizure states lie at the lower end. The Seizure state, after amplitude-guard correction, falls below Sleep—consistent with the hypersynchronous, low-dimensional dynamics associated with seizures (Jiruska et al. 2013). We emphasise that ictal phenomenology is heterogeneous—focal aware seizures can preserve consciousness—and that our generative model targets the specific class of hypersynchronous generalised seizures, not seizures in general; the low Ψ value obtained here should therefore be read accordingly. Decomposing Ψ into its components provides mechanistic interpretation, i.e., scale-free temporal organisation distinguishes disorganised noise from structured fluctuations, organised cross-frequency complexity captures structured phase–amplitude interactions, and metastability reflects the integration–segregation balance (Canolty and Knight 2010; Tognoli and Kelso 2014; Hancock et al. 2023). On the synthetic side the discriminative range of *D* derives largely from the Seizure state, where the amplitude guard sets D=0, together with the min–max normalisation step, rather than from graded differences across the consciousness hierarchy, where raw *D* is nearly constant across non-seizure states.

The Sleep-EDF application produces a Wake > (N2, REM) ordering with full paired consistency: rank-biserial $r_{rb} = +1.00$ on both Wake-versus-sleep contrasts. Every one of the 30 subjects ranks Wake above each sleep stage. The N2-versus-REM contrast is only weakly discriminated (p=0.058), reflecting the known difficulty of separating these two low-vigilance states on scalp EEG. The benchmarking result bears careful reading. Established complexity baselines (LZC, spectral slope, alpha power) all rank REM above N2 on this contrast because REM EEG is genuinely more complex than NREM EEG, and this ordering is arguably correct if the target is phenomenal richness (REM dreaming involves vivid experience). Under a vigilance construct, however, N2 above REM is the expected ordering, and the baselines reverse it. The composite’s nominal N2-above-REM direction follows from its per-subject Wake calibration of $H_{\textrm{opt}}$ rather than from any reliable separation, since on this contrast the composite and all three components sit close to chance (Höhn et al. 2024; Lendner et al. 2020; Brake et al. 2024; Maschke et al. 2024). We therefore treat the within-sleep direction as unresolved and as consistent with two readings at once, namely that N2 and REM may be genuinely close in consciousness-related organisation and that two channels may be insufficient to separate them. More generally, the framework behaves differently in the synthetic and the two-channel empirical settings, with $H_{\textrm{eff}}$ dominating in the synthetic ensemble and the rank structure of the raw cross-frequency term carrying the real-EEG contrast. We read this as a property of the restricted two-channel recording rather than of conscious dynamics.

Conceptually, Ψ may be viewed as a spontaneous-activity complement to perturbational complexity measures such as PCI—the two approaches are related in motivation but distinct in what they measure and when they can be applied (Casali et al. 2013; Maschke et al. 2024). Unlike single-aspect indices (fractal scaling, entropy, PAC, or synchrony alone), Ψ integrates three complementary properties, which allows it to distinguish states with similar scaling structure but differing cross-frequency or metastable organisation.

### Limitations and future directions

Several limitations should be kept in mind. Most of the results are obtained from synthetic data generated by a stylised EEG-like model that lacks neuronal microcircuitry and anatomical detail. It reproduces the macro-level dynamical features needed to validate the framework but should not be read as a biophysical model. The mapping between simulated and real states is approximate (e.g., the “psychedelic” state does not model receptor pharmacology).

The empirical application is restricted to two-channel polysomnographic recordings and to the Wake, N2, and REM stages. Two channels reduce metastability to a pairwise phase-lag measure and do not reliably resolve theta–gamma PAC (surrogate *z*-scores ∈[-0.03,0.15]), so the discriminative signal on this dataset is carried primarily by $H_{\textrm{eff}}$ and the subject-level rank structure of *D*, which on these two-channel data reduces to the raw theta–gamma index $I_{\phi A}$. The N2-versus-REM contrast sits at the edge of statistical significance. Larger samples or higher-density EEG are needed to resolve it robustly. The Wake-referenced calibration of $H_{\textrm{opt}}$ is a within-subject, within-cohort domain adaptation rather than external held-out validation. The anchor $H_{\textrm{opt}}^{\textrm{subj}}$ is estimated from Wake epochs, which are one side of the Wake-versus-sleep comparisons, and the scale term $\Delta \alpha ^{\textrm{subj}}$ uses the empirical $\bar{\alpha }_{\textrm{dfa}}$ range across all retained stages for that subject (thus including the evaluated sleep stages). We report this dependence transparently. Because $H_{\textrm{eff}}$ peaks at each subject’s own Wake median by construction, Wake is advantaged on this component, so the strong Wake-versus-sleep separation should be read partly as a property of the calibration design. The N2-versus-REM comparison, which does not include Wake among the stages being compared, is the more stringent anchor-free test within the dataset and it remains weak. Cross-cohort calibration with a fixed $H_{\textrm{opt}}$, evaluated on an external cohort never used for calibration, is a natural next step.

The most critical future direction is validation on higher-density, multi-state recordings, including anaesthesia induction and emergence, psychedelic states, and disorders of consciousness, as well as NREM stages not covered here (N1 and N3). Direct comparisons with established metrics such as PCI, permutation entropy, and connectivity indices will clarify whether Ψ offers complementary or superior discriminative power (Sitt et al. 2014; Melloni et al. 2023; Doerig et al. 2021; Luppi et al. 2024; Ferrante et al. 2025). Alternative choices for each component—different DFA parameterisations, more sophisticated PAC estimators (Castillo-Barnes et al. 2025; Aru et al. 2015), or alternative metastability indices (Hancock et al. 2025)—also merit systematic comparison.

A further consideration is volume conduction. The two-channel Sleep-EDF analysis uses the montage supplied by PhysioNet (Fpz-Cz and Pz-Oz, referenced to the contralateral mastoid); alpha-band phase synchrony in surface EEG is known to be contaminated by volume-conduction effects that inflate zero-lag synchrony (Aru et al. 2015). At C=2, the Kuramoto order parameter reduces to a pairwise measure, so this confound is absorbed into the *M* estimate. We do not apply a phase-lag correction in the present analysis; phase-lag-based synchrony estimators (e.g., weighted phase-lag index) and higher-density montages are the appropriate settings in which to revisit *M*, and are flagged as future work.

Finally, we emphasise that the synthetic and real-EEG analyses instantiate the same conceptual framework but not identical numerical operators. The synthetic *D* uses a 30–80 Hz gamma band; the real *D* uses 30–45 Hz, reflecting the Nyquist constraint of 100 Hz recordings. The synthetic $H_{\textrm{eff}}$ uses a Gaussian centred at $H_{\textrm{opt}} = 0.35$; the real $H_{\textrm{eff}}$ uses a per-subject Wake-anchored triangular mapping. The synthetic *M* is estimated from 16 channels, whereas the real *M* is estimated from two channels. Synthetic and real Ψ values are therefore not directly numerically comparable and should not be treated as interchangeable. What is preserved across both settings is the pipeline structure—three components, reference normalisation, weighted sum—and the qualitative claim that the composite captures organised complexity rather than raw complexity.

## Conclusion

We have presented a composite framework that combines three complementary properties of neural dynamics, i.e., scale-free temporal organisation, cross-frequency organisation, and metastability, into a single index Ψ. The motivation for this approach is that consciousness-related brain activity is unlikely to be captured by any single dynamical property alone. Rather than equating consciousness with greater complexity in a generic sense, the proposed framework treats conscious brain dynamics as organised complexity, i.e., activity that is temporally structured, functionally coordinated across frequencies, and flexible enough to move between integrated and differentiated network states.

In a synthetic EEG-like generative model, the index reproduces the qualitative ordering of nine brain-state classes, and systematic ablation and sensitivity analyses confirm that each component is non-substitutable and that the chosen hyperparameters sit on stable plateaus of the parameter space. Applied to two-channel Sleep-EDF recordings from 30 participants, Ψ separates wakefulness from both N2 and REM sleep at the subject level with every subject ranking wakefulness higher. Neither the composite nor the established baselines reliably separate N2 from REM. These empirical results should be interpreted as proof-of-principle evidence rather than clinical validation. The Sleep-EDF analysis demonstrates that the framework can generalise beyond the synthetic setting and can avoid a known failure mode of one-dimensional complexity measures, but the use of two-channel polysomnography necessarily limits the estimation of cross-frequency organisation and metastability.

The strongest claim here is not that Ψ measures consciousness directly, but that the three-component dynamical composite can separate wake from sleep at the subject level, while the within-sleep N2-versus-REM direction remains unresolved on these data. More broadly, the results support the view that vigilance-relevant neural dynamics are better characterised by the organisation of complexity than by raw complexity alone. Full validation across higher-density EEG, anaesthesia, and disorders-of-consciousness cohorts remains the essential next step.

## Data Availability

The code to compute the consciousness index Ψ, including the feature pipeline, the synthetic EEG generator, and validation scripts, is publicly available in a dedicated GitHub repository: https://github.com/ugail/Index-for-Consciousness-Dynamics. The real EEG recordings used for validation were obtained from the publicly available Sleep-EDF “Expanded” dataset, hosted on PhysioNet (https://physionet.org/content/sleep-edfx/). This dataset is distributed under the Open PhysioNet License. For full reproducibility and independent verification, the code for all experiments, including the ablation, sensitivity, Monte Carlo, and subject-level Sleep-EDF validation analyses, is available in the GitHub repository. The released code includes the full state-wise simulator configuration used for all synthetic analyses, including band weights, coupling constants, PAC strengths, noise parameters, slow-drift settings, and seizure-burst parameters.
